# Patient, Caregiver, and Decliner Perspectives on Whether to Enroll in Adaptive Deep Brain Stimulation Research

**DOI:** 10.3389/fnins.2021.734182

**Published:** 2021-10-07

**Authors:** Simon Outram, Katrina A. Muñoz, Kristin Kostick-Quenet, Clarissa E. Sanchez, Lavina Kalwani, Richa Lavingia, Laura Torgerson, Demetrio Sierra-Mercado, Jill O. Robinson, Stacey Pereira, Barbara A. Koenig, Philip A. Starr, Aysegul Gunduz, Kelly D. Foote, Michael S. Okun, Wayne K. Goodman, Amy L. McGuire, Peter Zuk, Gabriel Lázaro-Muñoz

**Affiliations:** ^1^Program in Bioethics, University of California, San Francisco, San Francisco, CA, United States; ^2^Center for Medical Ethics and Health Policy, Baylor College of Medicine, Houston, TX, United States; ^3^Baylor College of Medicine, Houston, TX, United States; ^4^Department of Anatomy and Neurobiology, School of Medicine, University of Puerto Rico, San Juan, Puerto Rico; ^5^Department of Neurosurgery, University of California, San Francisco, San Francisco, CA, United States; ^6^Fixel Institute for Neurological Diseases, Program for Movement Disorders and Neurorestoration, Department of Neurology, University of Florida, Gainesville, FL, United States; ^7^Department of Biomedical Engineering, University of Florida, Gainesville, FL, United States; ^8^Department of Psychiatry and Behavioral Sciences, Baylor College of Medicine, Houston, TX, United States

**Keywords:** aDBS, altruism, decision-making, interviews, quality of life, research, study design, symptoms

## Abstract

This research study provides patient and caregiver perspectives as to whether or not to undergo adaptive deep brain stimulation (aDBS) research. A total of 51 interviews were conducted in a multi-site study including patients undergoing aDBS and their respective caregivers along with persons declining aDBS. Reasons highlighted for undergoing aDBS included hopes for symptom alleviation, declining quality of life, desirability of being in research, and altruism. The primary reasons for not undergoing aDBS issues were practical rather than specific to aDBS technology, although some persons highlighted a desire to not be the first to trial the new technology. These themes are discussed in the context of “push” factors wherein any form of surgical intervention is preferable to none and “pull” factors wherein opportunities to contribute to science combine with hopes and/or expectations for the alleviation of symptoms. We highlight the significance of study design in decision making. aDBS is an innovative technology and not a completely new technology. Many participants expressed value in being part of research as an important consideration. We suggest that there are important implications when comparing patient perspectives vs. theoretical perspectives on the choice for or against aDBS. Additionally, it will be important how we communicate with patients especially in reference to the complexity of study design. Ultimately, this study reveals that there are benefits and potential risks when choosing a research study that involves implantation of a medical device.

## Introduction

Deep brain stimulation (DBS) is a well-established neurosurgical procedure whereby electrodes are surgically implanted into the brain to address common motor symptoms associated with movement disorders. For just over two decades, DBS has had remarkable success in the alleviation of select symptoms relating to Parkinson’s disease (PD) and has become an important therapy for motor symptoms and movement disorders (Hariz, 2017; Hartmann et al., 2019; Artusi et al., 2020). Given the relative success of DBS, there has been considerable interest in whether DBS might be used for treating other brain disorders including Alzheimer’s disease, obsessive compulsive disorder (OCD), and Tourette syndrome. A body of literature has emerged exploring both the likely efficacy and the ethical implications of a broader application of DBS, especially focusing upon the medical and ethical implications of addressing psychological symptomologies as opposed to motor symptoms (Widge et al., 2016a,b; Siegel et al., 2017; Aldehri et al., 2018; Lawrence et al., 2018; Viaña and Gilbert, 2019; Vicheva et al., 2020; Xu et al., 2020; Bonomo and Vetrano, 2021; Smith et al., 2021).

In addition to potentially broadening the scope of DBS usage, an innovative form of DBS is being trialed—known as adaptive deep brain stimulation (aDBS) or closed loop DBS—wherein a sensor electrode is employed to track fluctuations in brain activity possibly associated with clinical symptoms and the system is programmed to deploy or to adjust the level of stimulation (Habets et al., 2018; Swann et al., 2018; Little and Brown, 2020). In part, the development of aDBS was in response to some of the side effects of conventional DBS, including dysarthria, imbalance, hypomania, and dyskinesia as there was an inability to reduce these side effects by adjusting stimulation in real time (Widge et al., 2016b; Habets et al., 2018). By addressing this limitation, aDBS theoretically would be able to provide a more personalized or tailored program of stimulation, thus reducing the likelihood of over- or under-stimulation. Moreover, since this strategy does not employ continuous stimulation, aDBS technology has the potential to increase battery life, decreasing surgeries, and thus reducing morbidities associated with battery replacement surgeries. aDBS does, however, raise philosophical questions about the capacity of the device to stimulate or not stimulate without human input. As Klein et al. (2016) have penned, aDBS is a distinctly novel form of neuromodulation by which “one has effectively constructed a device that autonomously determines what the patient may or may not feel.” This has raised concerns about the impact of aDBS on personal autonomy given that that the algorithm self-directs the stimulation (Goering et al., 2017; Lázaro-Muñoz et al., 2017; Kostick and Lázaro-Muñoz, 2021).

These philosophical concerns may or may not play a role in the decision-making process of prospective DBS surgery patients. Individuals considering aDBS must decide (i) whether they feel brain surgery of any type is their best option, (ii) whether they feel that experimental aDBS is preferable to standard—non-adaptive—DBS (if available for their condition), and (iii) whether they want to be part of a research study (given that aDBS is only offered as part of research rather than as standard clinical practice). The following manuscript provides empirical data on how patients and caregivers reach a decision on accepting or declining aDBS surgery for the alleviation of the symptoms of dystonia, OCD, essential tremor, PD, and Tourette syndrome.

## Methods and Analysis

This study was embedded into aDBS clinical trials at Baylor College of Medicine, UCSF, and the University of Florida. Clinicaltrial.gov numbers are as follows: Baylor College of Medicine—NCT03457675, NCT04281134; UCSF—NCT03131817, NCT01934296, and NCT03582891; University of Florida—NCT02649166, NCT02056873. Semi-structured interviews were conducted with trial patients and their caregivers using a similar set of questions, both prior to surgery and approximately 6 months post-surgery. Individuals who decided not to participate in the aDBS trial were interviewed at one time point post-decline. Interviews were conducted in person or *via* Zoom/phone and lasted an average of 30–35 min for patients and caregivers and 20–25 min for decliners. The interviews were audio recorded and transcribed verbatim, with consent. All transcripts were de-identified prior to analysis. This study was approved by the Baylor College of Medicine Institutional Review Board.

Each interview cohort (patients, patient caregivers, and study decliners) was asked about the decision to participate or not to participate in a trial which would implant aDBS: Why did you decide to enroll in the study? (patients); what do you think about the patient’s decision to enroll in the study (patient caregivers); why did you decide not to enroll in the study? (study decliners). To identify patient responses to these questions, two members of the research team independently coded each interview transcript using MAXQDA 2018 qualitative data analysis software (Kuckartz). In line with established principles of qualitative research, we conducted interviews until reaching theme saturation, understood as a point at which interviewees were no longer raising novel themes relative to previous interviewees (Saunders et al., 2018). These text segments were then progressively abstracted (SO) utilizing thematic content analysis to identity a set of themes and sub-themes, which were corroborated (KM). The frequencies were not intended to suggest any level of statistical significance but were treated as descriptive data detailing how often a particular theme emerged organically and/or in response to interview questions.

## Results

Response rate were as follows: Response rates: 21 out of 23 patients = 91.3%, 20 out of 20 caregivers, and 10 out of 14 decliners = 71.4%. Of the 21 patients who agreed to interview, all but one had a respective caregiver who was also interviewed. One patient did not feel there was anyone who counted as being in a caregiver role for them. Demographic variables are provided to indicate the characteristics of the population interviewed (see Tables 1–3). Although data was reviewed to see if there were any immediately evident differences in population characteristics between patients and decliners, the small size of the population precluded further detailed statistical analysis.

**TABLE 1:** Patient demographics^†^.

Variable	Group					
	**OCD**	**Essential tremor**	**Tourette syndrome**	**Parkinson’s**	**Dystonia**	**Total**
	***n* = 5**	***n* = 3**	***n* = 4^∗^**	***n* = 8**	***n* = 1**	***n* = 21^∗^**
**Age**						
Mean	35.6	71.3	32.5	54.6	57	50.1
Min-max	31–40	71–72	24–41	28–71	–	24–72
**How do you describe your gender?**						
Male	2 (40%)	2 (66.7%)	1 (50%)	6 (75%)	–	11 (57.9%)
Female	3 (60%)	1 (33.3%)	1 (50%)	2 (25%)	1 (100%)	8 (42.1%)
**Are you of Hispanic, Latino, or Spanish origin?**						
Yes	1 (20%)	–	–	3 (37.5%)	–	4 (21.1%)
No	3 (60%)	3 (100%)	2 (100%)	5 (62.5%)	1 (100%)	14 (73.7%)
No response	1 (20%)	–	–	–	–	1 (5.3%)
Race						
Asian	–	–	–	1 (12.5%)	–	1 (5.3%)
White	4 (80%)	3 (100%)	2 (100%)	6 (75%)	1 (100%)	15 (78.9%)
Other	1 (20%)	–	–	1 (12.5%)	–	3 (15.8%)
**Total household income (before taxes) from all sources in the last year**						
0 to $49,999	2 (40%)	1 (33.3%)	1 (50%)	2 (25%)	–	6 (31.6%)
50,000 to $99,999	2 (40%)	–	–	1 (12.5%)	–	3 (15.8%)
100,000 to $149,999	1 (20%)	1 (33.3%)	1 (50%)	2 (25%)	–	5 (26.3%)
150,000 or more	–	1 (33.3%)	–	3 (37.5%)	1 (100%)	5 (26.3%)
**Source of health insurance^∗∗^**						
Employer	–	–	–	1 (12.5%)	–	1 (5.3%)
Parents or partner	3 (60%)	–	1 (50%)	1 (12.5%)	1 (100%)	6 (31.6%)
Medicaid or other state insurance	2 (40%)	–	–	3 (37.5%)	–	5 (26.3%)
Medicare	–	3 (100%)	1 (50%)	2 (25%)	–	6 (31.6%)
Private health insurance	–	–	–	1 (12.5%)	–	1 (5.3%)
Other	–	–	1 (50%)	–	–	1 (5.3%)

*^†^All patients with essential tremor, Tourette syndrome, Parkinson’s disease, and dystonia had additional implanted hardware and no patients with OCD had additional hardware. The additional hardware was placement of cortical strips in addition to the DBS leads.*

*Two patients receiving aDBS for Tourette syndrome did not complete the demographics survey. All “Total” percentages are therefore calculated with *n* = 19 as a denominator and within-disorder percentages for Tourette syndrome calculated with *n* = 2 as a denominator.

**Sums to greater than 100% because patients were able to select multiple options.

**TABLE 2:** Caregiver demographics.

**Variable**	**Group**				
	**OCD**	**Essential Tremor**	**Tourette Syndrome**	**Parkinson’s Disease**	**Total**
	***n* = 5**	***n* = 3**	*n* = 4*	***n* = 8**	*n* = 20*
**Age**					
Mean	52	69	48.3	57.8	56.5
Min-max	30–70	65–73	23–68	31–72	23–73
**How do you describe your gender?**					
Female	3 (60%)	2 (66.7%)	3 (100%)	6 (75%)	14 (73.7%)
Male	2 (40%)	1 (33.3%)	–	2 (25%)	5 (26.3%)
**Are you of Hispanic, Latino, or Spanish origin? (*n* = 18)^∗∗^**					
Yes	1 (20%)	–	–	1 (14.3%)	2 (11.1%)
No	4 (80%)	3 (100%)	3 (100%)	6 (85.7%)	16 (88.9%)
**How do you describe your race?^∗∗∗^**					
American Indian or Alaska Native	1 (20%)	–	–	1 (12.5%)	2 (10.5%)
White	4 (80%)	3 (100%)	3 (100%)	7 (87.5%)	17 (89.5%)
Other	1 (20%)	–	–	1 (12.5%)	2 (10.5%)
**What is your relationship to the patient?**					
Spouse	2 (40%)	3 (100%)	1 (33.3%)	8 (100%)	14 (73.7%)
Mother	3 (60%)	–	2 (66.7%)	–	5 (26.3%)

*^∗^One caregiver of a patient receiving aDBS for Tourette syndrome did not complete the demographics survey. All “Total” percentages are therefore calculated with *n* = 19 as a denominator and within-disorder percentages for Tourette syndrome calculated with *n* = 3 as a denominator.*

*^∗∗^One caregiver of a patient receiving aDBS for Parkinson’s disease completed other survey questions but did not respond to this question.*

*^∗∗∗^Sums to greater than 100% as respondents were asked to select all that apply.*

**TABLE 3:** Decliner demographics.

**Variable**	**Group**		
	**OCD**	**Parkinson’s**	**Total**
	**(*n* = 2)**	**(*n* = 8)**	**(*n* = 10)**
**Age (*n* = 9)^∗^**			
Mean	39	54.6	52.9
Min-max	–	38–68	38–68
**How do you describe your gender?**			
Female	1 (50%)	4 (50%)	5 (50%)
Male	1 (50%)	4 (50%)	5 (50%)
**Are you of Hispanic, Latino, or Spanish origin?**			
No	2 (100%)	8 (100%)	10 (100%)
Yes	–	–	–
**How do you describe your race?**			
Asian	–	1 (12.5%)	1 (10%)
White	2 (100%)	7 (87.5%)	9 (90%)
**Total household income (before taxes) from all sources in the last year (*n* = 9)^∗∗^**			
0 to $49,999	1 (50%)	–	1 (11.1%)
50,000 to $99,999	–	1 (14%)	1 (11.1%)
100,000 to $149,999	–	3 (43%)	3 (33.3%)
150,000 or more	1 (50%)	3 (43%)	4 (44.4%)
**Source of health insurance**			
Employer	1 (50%)	4 (50%)	5 (50%)
Parents or partner	–	1 (12.5%)	1 (10%)
Healthcare marketplace	–	1 (12.5%)	1 (10%)
Medicare	–	1 (12.5%)	1 (10%)
Private health insurance	1 (50%)	–	1 (10%)
Other	–	1 (12.5%)	1 (10%)

*^∗^One decliner of aDBS for OCD completed other survey questions but did not respond to this question.*

*^∗∗^One decliner of aDBS for Parkinson’s disease completed other survey questions but did not respond to this question.*

It may be of note that most decliners (7) were high-income (>$100,000), while just under half of patients were. Figures were too small to draw any conclusions from this difference. It should also be noted that income figures are difficult to compare due to major differences between states in respect to cost of living and average income. Nearly all patients received health care through either parents/partner (6), Medicaid (7), or Medicare (6). Only one patient received health care through their employer. By contrast, only half (5) of decliners received health care through their employer, only 1 through parent/partner, only 1 through Medicare, and 0 through Medicaid. Again, figures are too small to draw conclusions about these differences.

A condensed summary providing frequency of themes for undergoing aDBS is provided in Table 4, below.

**TABLE 4:** Relative frequency of themes for undergoing aDBS.

**Theme**	**Frequency (including patients and caregivers)**
Hopes for symptom alleviation	27
Declining or low quality of life	20
Altruistic motivations	12
Percieved benefits of being in research	11
Recommendations from others and trust in surgical team	9
Relatively low risk of aDBS	6
Benefits of new technology	5

### Patients’ and Caregivers’ Reasons for Trial Participation

#### Hopes for Symptom Alleviation Through aDBS

Hopes for symptom alleviation from aDBS surgery played a significant role in the decision-making process. In total, 27 out of 41 interviewees (66%) expressed hope for some form of benefit from aDBS. A greater percentage of patients shared that they were hopeful that the surgery would alleviate their symptoms (17/21, 81%) than caregivers (10/20, 50%). As Table 5 indicates, interviewees tended to see aDBS as a good option *given the circumstances*; they recognized that although it may not work, they felt there was nothing to lose at this point in their disease progression. However, even some patients who self-identified as reasonably healthy decided to enroll in an aDBS trial, hoping that it would prevent their symptoms from getting worse (see final quote in Table 5).

**TABLE 5:** Hope for improvement of symptoms.

It’s a good opportunity that she may not have otherwise had the chance to get, and I’ll take 75% odds of improvement any day of the week. [Caregiver]
I figured after about 13 years of dealing with this, anything’s worth trying at this point. [Patient]
So when we talked about it, when he was first offered the opportunity, we kind of were like, “Well, the worst that will happen is that it doesn’t work. And then you’re in no worse position than you are now.” [Caregiver]
There’s no guarantee. This may not work. It may not make her feel better, but it may. [Caregiver]
I will be really thankful to have a chance at feeling better, have a chance at being able to confront my OCD better and decrease some of those OCD symptoms. If that’s what happens, that obviously would be amazing. [Patient]
I still drive motorcycles and cars and we eat out. But if I didn’t do anything, it would change because I would feel really bad if she had to feed me. I wouldn’t like that. [Patient]

#### Quality of Life

Relatedly, about half of the interviewees (20/41, 49%) reported a decline in quality of life as a reason for trial participation, with 14/21 (67%) patients and 6/20 (30%) caregivers raising quality of life issues as a rationale for choosing to have aDBS surgery (see Table 6, below).

**TABLE 6:** Quality of life.

And the one thing I really want to get, my goal is to get my tics under control enough to where I can drive to and from work and pick up full term hours again. [Patient]
[W]e feel like it’s developed into what it’s developed into, which I think has put us in a lot more severe category of OCD and what she’s dealing with, which is, luckily, like I said, we stumbled across this because it seems like a potential solution to be able to help her gain some of that control back in her life and develop the skills and techniques to be able to move forward after this. [Caregiver]
I know she sits there day in, day out and maybe thinks about it all the time, but her life has passed her by. She rarely leaves the house and when she does it’s extremely difficult. [Caregiver]
It’s worse now than it’s ever been, especially in the past three, four years that I’ve known him. The past three years that I’ve lived with him. I know at this point, daily life has become a huge struggle for him, just on the basis of how severe his Tourette’s has become. [Caregiver]
[A]fter knowing her and going through treatment and trying different medications, and those types of things, finding this was what we felt as a last option to really be able to hopefully get her some long-term long lasting relief. [Caregiver]
This seems like a good time because I am trying to get lots done and I could have a, I’m trying to get lots done in my life, like I’m not retired or anything. So the intention was to get it early while the surgery is low risk on me as a person, and there’s a good potential for increased quality of life for many years. [Patient]
I feel like I have several medicines but none of them works. [Caregiver]
I am sort of a different case because I am so young. So I kind of see it as I have an opportunity to do something that could benefit me more for a longer period of time. [Patient]

#### The Desirability of Being in Research

A number of patients 11/41 (27%; including nine patients and two caregivers) referred to being in a research trial—rather than specific benefits of aDBS—as being an important consideration in choosing to enter the respective study. These considerations were largely centered upon the benefits of being closely monitored as a research patient and a generalizable technological enthusiasm. Finally, receiving treatment free of charge due to being in research was specifically raised by two patients (see Table 7, below).

**TABLE 7:** Benefits of being in research.

I talked to a couple people about it, and they said that you got a lot more access to the research team and you get a lot better care and you’re monitored much closer. [Patient]
First of all, I think he’ll get more attention. Yeah, he’ll be looked after better because he’s got a lot of people following this and checking up on us and checking on him. [Caregiver]
I think it’s wonderful. I think joining the study is probably going to give her better care in the long run than she would have had if she didn’t join the study. There’d be more follow-up appointments, more tweaking of the monitors and this kind of stuff. [Caregiver]
Well, it just sounded intriguing to me. It just sounded as if it was one step further, one step better than just the old normal kind of DBS. [Patient]
I should be part of the newest, latest, and greatest that they have available. And so that’s kind of one reason. [Patient]
I just completely given up, on getting better. Cause I’m never, unless I win Power ball, or something or somebody wants to just generously pay for my surgery, it would never happen. So, I was excited when I came upon this study. [Patient]

#### Altruism and Helping Oneself

Altruism was another of the more frequently occurring rationales for electing to participate in an aDBS trial, with 12/41 (29%) of interviewees referring to one or more forms of altruistic rationale. As summarized in Table 8, the expression of altruism varied significantly, from hoping to contribute to science, to helping others with the same condition, to potentially helping future patients or family members. Often, the distinction between each was blurred or different expressions of altruism were referred to by the same interviewee. It is notable that altruism was expressed far more often by patients (11/21) than caregivers (1/20), suggesting patients might see themselves as directly giving or contributing more often than caregivers. It is possible that patients felt more justified in deciding to undergo brain surgery on themselves for altruistic reasons, while caregivers primarily thought about benefits and risks to the patient rather than the general population (see Table 8, below).

**TABLE 8:** Expressions of altruism.

We wish he could participate in more studies, but it’s hard to get involved – so it was nice that there was one that might help the science, and that he qualified for. [Caregiver]
[I]f this can help others or science, I figure I’m going to have the deep brain DBS surgery. [Patient]
I can’t imagine how many other people are suffering in this way or in severe ways too, and how debilitating it is. If there’s anything that I can do on that other end to help while I’m still doing the surgery that I want to do to help myself, if I can add on a little bit that could possibly help down the road for myself or other people, great. That to me is worth it. [Patient]
I talked to her [patient’s daughter] just a few minutes ago about it and she asked me everything that went on today and I told her and she knows that someday she may have to go through this same thing. [Patient]

It is important to note that for several interviewees, altruism was not an exclusive motivation but instead was combined with the hope that the technology would improve their own lives, as illustrated in the following quotes:

From a selfish standpoint I want to try it, but also from a standpoint of helping me, helping the research, I felt that it would be good to participate. [Patient]
If I can add on a little bit that could possibly help down the road for myself or other people, great. That to me is worth it. [Patient]
At first, I really liked the idea that it could help further OCD research, just that of course, first and foremost, I will be really thankful to have a chance at feeling better. [Patient]

#### Recommendations From Others

Although perhaps relatively non-controversial, an important consideration among interviewees was the advice of others that undertaking to have aDBS would be a good choice. This particularly related to advice from a patient’s primary care provider. A total of nine interviewees (22%) including seven patients and two caregivers indicated that this was important in their decision making. Table 9 provides illustrative excerpts from interviews on this subject.

**TABLE 9:** Recommendations from others.

I had been seeing Dr. [anonymous] for almost 9 years, and last year around [month] he mentioned the DBS study and he thought that I would be a good candidate, so I decided to participate.[Patient]
My sister knew a guy that had it. And she said that her friend said it was one of the best things he’s ever done in his life and I started looking into it and I started reading about it. Then I looked up the, the, I looked up the, I found a rating of places that have it done, and I thought this is the top one in [location ananymous]. [Patient]
He [patient’s doctor] said, “You know,” he said, “I’m out of options as far as medication.” He’s like, “We did them all.” He said, “We just can’t give you more strength of what you’ve got going.” He says, “I recommend you go see them about getting the surgery.” We did. [Patient]

### Study Decliners’ Reasons for Not Enrolling in an aDBS Trial

Study decliners provided a range of rationales for why they chose to decline aDBS (Table 10). The primary rationale for declining was practical, with 7 out of 10 (70%) highlighting that they declined aDBS because the study was located inconveniently, involved too much time in follow-up, or was not scheduled in manner that was convenient to them. Other factors were more specific and include not being able to have an MRI (20% of decliners), people seeing the wires (20% of decliners), and concerns over health insurance (20%).

**TABLE 10:** Specific reasons not to have aDBS.

Practical issues
Well, it’s mainly logistics because I live in [STATE 1] and it’d be hard to keep up with it from [STATE 2] and all that. And that was a big reason for it.
[I]f I had to wait until May or June and then maybe it didn’t work I would kind of kick myself in the butt, like, “Why didn’t you just do the old one, [NAME], and have higher chance of success, you know?”
Not being able to have an MRI in the future
[T]here are two other reasons I think that almost took precedent. One was the effect that I may not be able to get another MRI for the rest of my life, and that actually troubles me because I have some other issues that may require a surgery and therefore the consideration of an MRI. One is the stenosis of the cervical spine.
Cosmetic/Noticeable wires
Maybe vanity, you know? Just people thinking, talking, “Well, why does [Respondent Name] have two wires sticking out of his head?” Just that. You know? Again, I go back to the fact that, okay, if I was severe, I really wouldn’t care what people say.
Impact on health insurance (*n* = 2/10 decliners)
[P]otentially losing any insurance in the future because of a complication from a research device. You know I just – I wouldn’t know where that’s gonna go until it’s – maybe some mandates in place to say you know, insurance your gonna pay for this.

### Balancing the Risks and Benefits of aDBS Surgery as Novel Technology

The status of aDBS as a novel form of technology was a key consideration for both patients and individuals declining aDBS. Patients tended to view the novelty of the technology as a positive, or opportunity to offer a more personalized and improved treatment system (newer = better), while study decliners saw it more negatively, as simply too experimental and risky (hesitancy to be among the first). It may be interesting to highlight that of total number of patients and caregivers interviewed, only 5 out of 41 (12%) referred specifically to the new technology as reason for choosing to have aDBS. A far greater percentage, 5 out of 10 decliners (50%) referred to concerns over new technology as one of the reasons for not undertaking to have aDBS (see Table 11, below).

**TABLE 11:** Evaluation of aDBS’s novelty.

Positive evaluation
[T]here is the potential to have it be a lot more specialized to the individual.
[T]hey can fine tune it a lot better than you can with just the old style.
Negative evaluation
My 21 year old daughter said to me, you can be the fifth person, you can be the sixth person, you can be the tenth person, but you will not be one of the first people.
This was like, “Well, you know, it’s starting as a study, and we haven’t put it many people,” and I was just not ready to go there yet.
I didn’t really have any main concerns, other than the fact that it was new.

Another theme that emerged was how for several participants aDBS was considered a relatively low additional risk in comparison to DBS. Six interviewees (five study participants and two caregivers) referred to their decision being influenced by this perception of relatively minimal extra risk of having aDBS, as compared to conventional DBS (see Table 12). It was notable that of the six interviewees referring to this issue, all but one were from the PD cohort.

**TABLE 12:** Additional risk of aDBS over conventional DBS.

It’s not the kind of trial where you have some people on a placebo and some people on the drug, so some people benefit. So, the way that I look at it is like it seems that I can pretty much just benefit. I mean, I consider the risks to be relatively low. [Patient]
I could always go to the tried and true so I could kind of have the option that they already have, which is the standard, I guess. [Patient]
I think I would have done deep brain stimulation even if it wasn’t in the adaptive. I think the adapter was an added bonus for me. [Patient]

## Discussion

Of those issues considered important in decision-making by persons who enrolled in aDBS, hopes for alleviation of symptoms and declining quality of life often arose together as reasons to have aDBS surgery. Another of the more frequently cited rationales for having aDBS was that being in research would provide access to a team of experts that might not otherwise be made available in standard clinical practice. Altruism also featured as an important rationale and was often combined with a hope that surgery might benefit the patient. Finally, personal recommendations from physicians or friends featured reasons to undergo aDBS surgery. Persons declining aDBS cited inconvenience, time commitments, and concerns about health insurance coverage as reasons not to undertake aDBS surgery. Finally, it was notable how relative risk featured in these deliberations in respect to the technology on offer. For some, the offer of a novel, experimental technology was considered a positive attribute and thus inclined them toward choosing aDBS. For others, the experimental nature of the technology concerned them and was a reason (among others) not to have aDBS. For a third group, the additional risk of being part of trial was counterbalanced by the knowledge that the adaptive element of aDBS, if ineffective, could be de-activated, allowing them to revert to standard DBS technology; as such, they saw the additional risk of aDBS over DBS as minimal compared to the overall risk of brain surgery itself. Notably absent from the decision making process was either an explicit enthusiasm or concern regarding the automaticity of the adaptive system. This is interesting both because automaticity (i.e., automatic change in stimulation using aDBS) has been raised in respect to potential concerns about aDBS (as seen above—Goering et al., 2017; Lázaro-Muñoz et al., 2017; Kostick and Lázaro-Muñoz, 2021) and that trust in automaticity has been seen as important factor in public enthusiasm (or otherwise) for other forms of automated technology such as automated cars (Jian et al., 2000; Hoff and Bashir, 2015; Schaefer et al., 2016). In part, this may be an artifact of the study question design wherein the purpose was to find the primary reason or reasons for entry into the study or non-enrollment. One could speculate that the enthusiasm or distrust of automaticity might still be part of the decision making process, but it did not appear high on agenda when considering the decision to have aDSB or decline aDBS. In a study being conducted at present, the research team is exploring interviewees’ understandings of the specific features of aDBS including automatic stimulation.

This study provides much needed empirical data on reasons for and against electing to surgically implant a neural device that is both innovative and potential ethically complex due to software algorithm-based control of an individual’s brain stimulation treatment in real time. It highlights the depth and breadth of considerations made in choosing whether to enter or decline aDBS research trials, and brings to the fore those elements of the decision that are important to persons themselves making this decision. These empirical decision-making factors include “push” factors wherein any form of surgical intervention is preferable to none and “pull” factors wherein opportunities to contribute to science combine with hopes and/or expectations for the alleviation of symptoms. Interviewee responses highlight that, regardless of whether they decided to participate in an aDBS trial or now, this was a decision made after considerable deliberation and often in consultation with others; as seen in respect to the number of people considering recommendations from others. No single issue determined whether someone elected to have aDBS or chose to decline. This depth of consideration reflects similar findings by Lawrence et al. (2018), who write in the context of conventional DBS that “participants seemed very aware of the risks, and very aware of their own difficulties processing information, and there was no indication that participants would make quick decisions to undergo DBS.”

Our findings suggest that those who chose to undergo aDBS often did so through either by reason of wanting to help others or a combination of altruism and a desire to help oneself. As similar complex interplay is found in a study by Locock and Smith (2011) in their own study of who takes part in such trials, whereby they conclude “While altruistic motivations were undoubtedly present in our participants’ decisions, potential personal benefit emerged as the prime motivator in this group of respondents. Where altruistic reasons were expressed, they tended to be in combination with personal reasons, or to be founded more on notions of ‘social exchange,’ than any purely selfless motivation.” In an additional complexity, altruism was expressed through a broad spectrum of rationales for having aDBS ranging from helping to forward scientific and human progress to a much closer identification of the community of persons suffering from the same or similar symptoms and even to personal identification of other persons who may benefit as a result of the individual joining the research. McCann et al. (2010) suggest that the term “conditional altruism” can be helpful when characterizing the decision to enter randomized control trials and “trial participation seemed to be something of a ‘win: win’ situation—one in which they [trial participants] could both help others and benefit (or at least not be harmed) personally.” The incorporation of scientific objectives as part of the individuals’ altruistic thinking may well have enabled the sort of win/win expectation, or at the very least one win out of a potential two.

The data also highlight the degree to which participants do not conceptualize their participation in terms of a clear boundary between research and clinical objectives. Rather than constituting a therapeutic misconception (see Appelbaum et al., 1987; McConville, 2017 for discussion of this term), we suggest that this blurred line of distinction reflects study designs wherein aDBS is an extension of existing technology that can be switched off if it proves to be ineffective or associated with undesirable side effects. Persons considering whether to have aDBS surgery as part of research weighed the advantages and disadvantages and chose accordingly; they did not deny the possibility of disadvantages in entering into the research process, but factored this into their decision-making. It is argued that interviewees were aware of the research vs. clinical distinction but, when choosing to undertake aDBS surgery, explicitly combined the scientific objective of creating generalizable knowledge with at least a hope that aDBS would alleviate their symptoms. In doing so, they blended the scientific objective—which some incorporated as their own motivation—with what might be referred to as qualified expectation of clinical benefit.

It is important to highlight—as some participants articulated—that if participant enrolls in the trial and gets aDBS, there still an option to turn off the adaptive component and treat them with conventional DBS. Mergenthaler et al. (2021) have referred to “opportunity studies” vs. “experimental trials.” In the former, there is likely to be only “a marginal increase in risk over the risk associated with the clinical intervention itself,” while in the case of experimental trials, investigators test devices as “stand-alone procedures” and subjects are “unlikely to receive neurosurgery if not for enrollment in research.” These offer very different contexts for choice and perceptions of risk. These considerations influence not only decision making about entering into a trial but also post-trial concerns about continued access to experimental technology or removal of such technology (the subject of a forthcoming manuscript by the team). In some cases, interviewees appear to express a clear understanding that they were entering into what Mergenthaler refers to as an “opportunity” study but it remains unclear as to the degree to which others fully understood this complex research/clinical relationship and the possibility of switching from aDBS to DBS.

One of the more notable expectations of aDBS was that rather than specifically alleviating symptoms, some saw aDBS surgery as an opportunity to maintain their existing quality of life by preventing decline, sometimes specifically identifying having a number of high-quality years still available to them if aDBS is successful (Schuepbach et al., 2019 for recent discussion of early stage intervention and quality of life). While these were patients with diagnosed disease who have symptoms and diagnosed condition, to a limited extent their comments raise a number of questions regarding the timing of surgical intervention. It is important to note that we do not have a clear understanding of whether DBS or aDBS can reverse symptoms for all conditions or slow the progression of the disease.

Finally, arguably, the most provocative and significant finding is that some people perceive the experimental aspect of their participation as positive (where they are first in line to receive a promising new treatment), while others perceive it as negative (that they are first in line to be “guinea pigs” for a treatment with potentially negative down-the-road consequences). As such, our findings suggest a strong need to re-address the theoretical perception of research as being largely one of additional risk, over and above clinical practice (see Lantos, 2014). As our interviews suggest, a considerable number of persons were drawn toward having aDBS because they felt that being part of research would be advantageous to them. In doing so, they often merged technological enthusiasm, wanting the best and newest technology, and an expectation of greater care and attention. These expectations are well worth considering in respect to broader literature on patient perspectives on entering into research (McCann et al., 2010, 2013; Locock and Smith, 2011; Jenkins et al., 2013; Hughes-Morley et al., 2015).

## Limitations

These in-depth interviews were intended to identify the range of responses that were offered by interviewees when discussing their choice to enter or decline aDBS trial participation. This approach is limited in the sense that it cannot provide generalizable results as it is restricted to these specific responses. Though we reached theme saturation in all three interviewee cohorts—participants, caregivers, and decliners—substantially more people interviewed were patients and respective caregivers, which biases our analysis to this perspective in respect to the depth of analysis.

## Conclusion

Our study highlights the requirement to improve our understanding of the considerations made in choosing whether to undergo aDBS surgery as part of research. Looking in detail at this choice making process is strongly suggestive of the need to reflect upon some of the more theoretically based concerns about aDBS raised and compare these to rationales expressed by patients, caregivers, and decliners about their choice to enter or decline aDBS. In addition, the study highlights the need for further research into how prospective study enrollees come to understand complex study protocols wherein an innovative technology is overlaid with an established clinical procedure. By improving our empirical knowledge of choice-making within this context, we should be able to improve our communication strategies, thus minimizing the likelihood of unwarranted expectations and misunderstandings of what is an inherently complex study protocol. Finally, in the light our findings that many participants viewed research as positive, the study highlights that we need to pay close attention to what patients believe are the benefits of being in research. In doing so, we can be better placed to make sure that such expectations are met.

## Data Availability Statement

The datasets presented in this article are not readily available as raw data will not be shared outside of the research team. Upon request, sections of the data may be provided for specific requests after the permissions of participants are requested and received.

## Ethics Statement

The studies involving human participants were reviewed and approved by the Baylor College of Medicine Institutional Review Board. Written informed consent for participation was not required for this study in accordance with the national legislation and the institutional requirements.

## Author Contributions

SO wrote the first draft. GL-M, PZ, AM, WG, BK, LT, SP, JR, and KK-Q contributed to conception and design. KM, KK-Q, CS, LK, RL, LT, DS-M, JR, BK, PS, AG, KF, MO, WG, AM, PZ, and GL-M contributed to recruitment and implementation. SO, KK-Q, KM, CS, LK, RL, LT, and PZ analyzed the data. PZ, KK-Q, AM, SP, and GL-M wrote sections of the manuscript. All authors contributed to manuscript revision and read and approved the submitted version of the manuscript.

## Conflict of Interest

The authors declare that the research was conducted in the absence of any commercial or financial relationships that could be construed as a potential conflict of interest. The handling editor declared a shared affiliation with three of the authors, SO, BK, and PS, at the time of review.

## Publisher’s Note

All claims expressed in this article are solely those of the authors and do not necessarily represent those of their affiliated organizations, or those of the publisher, the editors and the reviewers. Any product that may be evaluated in this article, or claim that may be made by its manufacturer, is not guaranteed or endorsed by the publisher.
